# *MTHFR* C677T polymorphism analysis: A simple, effective restriction enzyme‐based method improving previous protocols

**DOI:** 10.1002/mgg3.628

**Published:** 2019-03-13

**Authors:** Francesca Antonaros, Giulia Olivucci, Elena Cicchini, Giuseppe Ramacieri, Maria Chiara Pelleri, Lorenza Vitale, Pierluigi Strippoli, Chiara Locatelli, Guido Cocchi, Allison Piovesan, Maria Caracausi

**Affiliations:** ^1^ Unit of Histology, Embryology and Applied Biology, Department of Experimental, Diagnostic and Specialty Medicine (DIMES) University of Bologna Bologna Italy; ^2^ Neonatology Unit St. Orsola‐Malpighi Polyclinic Bologna Italy; ^3^ Neonatology Unit, Department of Medical and Surgical Sciences (DIMEC) St. Orsola‐Malpighi Polyclinic, University of Bologna Bologna Italy

**Keywords:** *MTHFR* C677T, new primer pair, PCR‐RFLP, risk factor, single nucleotide polymorphism

## Abstract

**Background:**

5,10‐Methylentetrahydrofolate reductase *(MTHFR)* C677T polymorphism is one of the most studied genetic variations in the human genome. Polymerase chain reaction‐restriction fragment length polymorphism (PCR‐RFLP) is one of the most used techniques to characterize the point mutations in genomic sequences because of its suitability and low cost. The most widely used method for the *MTHFR* C677T polymorphism characterization was developed by Frosst et al. (1995) but appears to have some technical limitations. The aim of this study was to propose a novel PCR‐RFLP method for the detection of this polymorphism.

**Methods:**

In order to retrieve all published articles possibly describing any PCR‐RFLP methods useful to analyze *MTHFR* C677T polymorphism, we performed systematic queries on PubMed, using a combination of Boolean operators (AND/OR) and MeSH terms. Amplify software was used in order to design a new primer pair following the optimal standard criteria. Primer‐BLAST software was used to check primer pair's biological specificity.

**Results:**

The analysis of previous literature showed that PCR‐RFLP method remains the most used technique. None of the 108 primer pairs described was ideal with regard to main accepted primer pair biochemical technical parameters. The new primer pair amplifies a DNA‐fragment of 513 base pair (bp) that, in the presence of the polymorphism, is cut by *Hin*f I enzyme in two pieces of 146 bp and 367 bp and clearly visible on 2% agarose gel. The level of expertise and the materials required are minimal and the protocol takes one day to carry out.

**Conclusion:**

Our original PCR‐RFLP strategy, specifically designed to make the analysis optimal with respect to PCR primers and gel analysis, fits the ideal criteria compared to the widely used strategy by Frosst et al (1995) as well as any other PCR‐RFLP strategies proposed for *MTHFR* C677T polymorphism genotyping to date.

## 1 INTRODUCTION

5,10‐Methylentetrahydrofolate reductase (*MTHFR*) gene (On‐line Mendelian Inheritance in Man ‐ OMIM accession number: 607093) is located on the short arm of chromosome 1 (1p36.22); it contains 13 exons and is 20,373 base pair (bp) long. The MTHFR enzyme plays a central role in folate and homocysteine metabolism by catalyzing the conversion of 5,10‐MTHF to 5‐MTHF (5‐Methyltetrahydrofolate), the primary circulatory form of folate which is utilized in homocysteine remethylation to methionine (Rosenblatt, 2001). Genetic variation in this gene influences susceptibility to occlusive vascular disease, neural tube defects, colon cancer, and acute leukemia; and mutations in this gene are associated with methylenetetrahydrofolate reductase deficiency (Schwahn & Rozen, 2001).

Two frequent polymorphisms in the human *MTHFR* gene confer moderate functional impairment of MTHFR activity for homozygous mutant individuals: the C677T and the A1298C. The C677T polymorphism has been extensively studied for its clinical consequences. It consists in nonsynonymous substitution (alanine to valine) on the catalytic domain that decreases in vivo enzyme activity by 35% in heterozygous subjects and by 70% in homozygous subjects (Frosst et al., 1995). The C677T numbering is based on the single nucleotide polymorphism (SNP) location attributed by Goyette and coll. (Goyette et al., 1994) even though the substitution is on exon 5 in position 894 of the current messenger RNA (mRNA) reference sequence (NM_005957.4).

The homozygous genotype for the polymorphism (TT) is particularly common (32% in Mexico, 26% in Southern Italy and 20% in Northern China) (Wilcken et al., 2003) and is correlated with many pathological risks (Liew & Gupta, 2015). In fact, the *MTHFR* C677T genotype was associated with an alteration in folate metabolism, DNA hypomethylation, and high homocysteine plasma level (DeVos et al., 2008). Folate deficiency in pregnant women is a risk factor for neural tube defects (Nauman et al., 2018) and congenital heart defects (CHD) (Wang et al., 2013) in the fetus. *MTHFR* C677T polymorphism is also considered a maternal risk factor for Down syndrome (DS) because it could promote abnormal chromosome segregation by compromising the availability of methyl groups (Rai, Yadav, Kumar, Yadav, & Mishra, 2014). The genetic cause of DS was discovered by Lejeune and coll. (Lejeune, Turpin, & Gautier, 1959) who identified a supernumerary chromosome 21 in the cells of subjects with DS. Recent works demonstrated the association of DS phenotype with a highly restricted Down syndrome critical region (HR‐DSCR) on human chromosome 21 (Pelleri et al., 2016) and with specific metabolic and gene expression alterations (Caracausi et al., 2018; Pelleri et al., 2018). DNA hypomethylation and folate deficiency might also result in malignancy transformation (Tang et al., 2014). Many studies show the association of C677T polymorphism with an increased risk for gastric cancer, breast cancer, and hepatocellular cancer in Asians and with gastric cancer, multiple myeloma, and non‐Hodgkin lymphoma (NHL) in Caucasians (Xie et al., 2015). A reduction in enzyme activity results in hyperhomocysteinemia (Fridman et al., 2008) and is considered an independent risk factor for cardiovascular disease, stroke, and venous thrombosis (Lippi & Plebani, 2012) and hypertension, including hypertension in pregnancy (Qian, Lu, Tan, Liu, & Lu, 2007). The *MTHFR* C677T was also significantly associated with psychiatric disorders such as depression, bipolar disorder (Gilbody, Lewis, & Lightfoot, 2007), and schizophrenia (Yadav, Kumar, Gupta, & Rai, 2016).

Polymerase chain reaction‐restriction fragment length polymorphism (PCR‐RFLP) is one of the most used techniques to characterize SNPs because of its suitability and low cost (Ota, Fukushima, Kulski, & Inoko, 2007). With this method, a DNA segment containing the mutation site is amplified by PCR and the presence/absence of the polymorphism is detected by loss or gain of a restriction site (Parsons & Heflich, 1997).

Despite *MTHFR* C677T polymorphism being one of the most studied genetic variations in the human genome (Christensen & Rozen, 2010), the most widely used method in literature to identify this polymorphism was developed by Frosst et al. (1995) and never carefully revised.

Many kinds of approaches were used (see Table S1) to study the *MTHFR* C677T polymorphism. In the present work, we describe a systematic bibliographic search about the polymorphism analysis which recovered 4,913 articles, 679 of them not presenting original data (Meta‐analyses, Reviews, or Comments including Hypothesis). The technique most used to analyze the *MTHFR* mutation was the PCR‐RFLP, described in 2,257 articles (1,803, i.e. 79.9%, explicitly using the primers from Frosst et al. (1995)). The second most technique used to study this mutation was Real‐time PCR (588 articles, 188 of them describing TaqMan SNP genotyping assay). All the other methods were described in a smaller number of articles, for example some authors used DNA sequencing with different approaches or SNaPshot for genotyping, which is the best solution for multiple‐SNPs association studies, or the DNA strip technology, but all of them are very expensive and require test‐specific technical expertise. All these methods and their references are available in the Table S1.

The method developed by Frosst et al. (1995) for the identification of the *MTHFR* C677T polymorphism appears to have some technical limitations. First of all, the binding site for the forward primer on the target sequence was extremely close to the polymorphism, so if a genotype confirmation by sequencing is desired, the electropherogram would not be readable for the first part where the SNP site is located. Furthermore, the forward primer overlaps with the recognition site for *Hin*f I and could hide potential mutations in the restriction site, leading to false results. Conversely, this is needed for the screening of methionine synthase reductase (*MTRR*) polymorphism A66G (I22M). In this case, the forward primer, overlying the cleavage site, generates a *Nde*I restriction site thanks to the substitution with A to C that generate a palindromic site in a normal sequence (Wilson et al., 1999). Finally, the digestion of PCR products with *Hin*f I restriction enzyme would generate one single band of 198 bp in nonmutated DNA and two bands (176 bp and 22 bp) if the mutation was present. This pattern makes it quite difficult to see the results on agarose gel: the 198 bp‐ and 176 bp‐ bands are barely distinguishable; the small 22 bp‐band could not be seen at all.

Following the original description of the method by Frosst et al. (1995), many other authors proposed different primer pairs to be used in PCR‐RFLP analysis for the screening of the *MTHFR* C677T polymorphism. Our systematic analysis retrieved 108 of these primer pairs, following examination of 4,913 articles related to the investigated polymorphism (Table S1). While the primer pair from Frosst et al. (1995) revealed to be by far the most used, we have been able to determine the usage frequency of all the other pairs and systematically compare the features of each previously used primer pair and ours. Results showed that none of these primer pairs was ideal with regard to main accepted biochemical technical parameters (Table S2).

The aim of this study was to implement the PCR‐RFLP analysis of *MTHFR* C677T polymorphism designing a new primer pair that allows us to obtain clearer results through an easier, safer, and more economical protocol, here described, in which polyacrylamide gel, which is toxic, is substituted with agarose gel and the forward primer does not overlap the polymorphism site. The primer pair was designed on introns 4 and 5 (NG_013351.1), and we used Amplify software (Engels, 1993) to test the primers on our DNA region of interest and to check the quality of primers with regard to potential secondary structure formation. Furthermore, we used Primer‐BLAST software (https://www.ncbi.nlm.nih.gov/tools/primer-blast/) to check for biological specificity. The primers were designed on a DNA region that did not contain any recognition site for *Hin*f I except for the one corresponding to the C677T mutation as we checked on EnzymeX 3.1 software for Mac OS X. The forward primer sequence is 5′‐TGTGGTCTCTTCATCCCTCGC‐3′; the reverse primer sequence is 5′‐CCTTTTGGTGATGCTTGTTGGC‐3′. The melting temperature (Tm) for both primers is 66°C and the annealing temperature (Ta) is 61°C. Guanine–cytosine (GC) content is 57% for forward primer and 50% for reverse primer. The primers do not show secondary structures and result specific when tested on Primer‐BLAST. The fragment amplified by PCR is 513 bp long. In Table 1 the main features of Frosst's primer pair and the novel one are compared.

**Table 1:** Comparison between Frosst and newly proposed primers’ characteristics

	Frosst et al., 1995		This work	
	Forward primer	Reverse primer	Forward primer	Reverse primer
Sequence (5′−3′)	TGAAGGAGAAGGTGTCTGCGGGA	AGGACGGTGCGGTGAGAGTG	TGTGGTCTCTTCATCCCTCGC	CCTTTTGGTGATGCTTGTTGGC
Tm	72°C	66°C	66°C	66°C
Tm difference (optimal <2°C)	6°C^a^		0°C	
Length (optimal 18−22 nt)	23	20	21	22
GC clamp	No^a^	No^a^	Yes	Yes
GC content (optimal 40%−60%)	56%	65%^a^	57%	50%
Secondary structures	No	No	No	No
Overlap between sense primer and *Hin*f I recognition site.	Yes^a^	Yes^a^	No	No
Simple visualization on agarose gel after RFLP	No^a^	No^a^	Yes	Yes
Presence of other restriction sites for *Hin*f I	No	No	No	No
Pairing specificity	Yes	Yes	Yes	Yes

a Not optimal parameter.

## 2 MATERIALS AND METHODS

### 2.1 Ethical compliance

Ethical approval for the study was granted by the Independent Ethics Committee of the Hospital‐University of Bologna Policlinico S. Orsola‐Malpighi, Italy (number:39/2013/U/Tess). Informed written consent for the collection of blood and clinical data as well as for genetic analysis was obtained from all patients or from parents if the patient was below 18 years of age.

### 2.2 Bibliographic search

In order to retrieve all published articles possibly describing any PCR‐RFLP methods useful to analyze *MTHFR* C677T polymorphism, we first performed systematic queries on PubMed at the NCBI site (http://www.ncbi.nlm.nih.gov/pubmed/), using a combination of Boolean operators (AND/OR) and MeSH terms. In particular, to also account for not standard methods referring to the polymorphism, we performed the following search: (MTHFR OR "Methylenetetrahydrofolate Reductase (NADPH2)"[Mesh] OR methylenetetrahydrofolate) AND (rs1801133 OR 677* OR C677* OR CT677* OR C>T677* OR MTHFR677* OR MTHFRC677* OR T677 OR ALA677* OR A222V OR ALA222VAL OR 667* OR C667* OR CT667* OR MTHFR667* OR MTHFRC667* OR T667 OR ALA667* OR thermolabil* OR "A/V" OR alanine‐to‐valine OR alanine OR valine) AND ("1995/01/01"[CRDAT]:"2018/04/18"[CRDAT]).

### 2.3 Sample selection

The study was proposed to subjects admitted to the Day Hospital of the Neonatology Unit, Sant'Orsola‐Malpighi Polyclinic, Bologna, in the context of the routine follow up provided for DS.

Twenty samples were chosen from a total of 146 genotyped samples, including 102 samples from DS subjects and 44 control samples retrieved from their non‐DS siblings. We selected 10 samples from the group of DS subjects and 10 samples from the control group. No specific algorithm was used, two independent experimenters simply picked random tubes from the storage box, ensuring that the three genotypes were represented in a similar proportion (CC = 7, TT = 7, and CT = 6).

We again picked three random samples among those assigned to each genotype to confirm the genotype by direct sequencing.

### 2.4 DNA extraction from peripheral blood

Blood samples were collected in ethylenediaminetetraacetic acid (EDTA)‐coated blood collection tubes and kept at room temperature; they were treated within 2 hr of blood draw. Genomic DNA was isolated from whole blood using QIAamp DNA Blood Midi Kit (Qiagen, Hilden, Germany) in accordance with the manufacturer's instructions. To obtain highly concentrated DNA, the eluate containing the DNA was reloaded on the membrane, incubated at room temperature for 5 min, then again centrifuged at 4,500 g for 2 min. DNA was eluted in 200 μl of distilled water and finally stored at −20°C until analysis.

In our study, genomic DNA was isolated from whole blood, but it can be obtained from different tissues (biopsies or saliva) and with different methods, such as DNA purification based on phenol extraction and ethanol precipitation (Moore, 2001) or commercially available DNA preparation kits.

### 2.5 Nanodrop DNA quantification

Absorbance‐based quantification of nucleic acid was obtained with NanoDrop‐1,000 Spectrophotometer (Thermo Scientific; Thermo Fisher Scientific, Waltham, MA) after pipetting 1 μl of each sample onto the end of the fiber optic cable. Duplicate analyses were performed on each sample, setting 0.2 mm pathlength to calculate the absorbance and the results were generated in ng/μl. Then the two values obtained for each sample were averaged. With this tool, nucleic acid samples can be checked for concentration and quality. The ratio of sample absorbance at 260 nm and 280 nm is used to assess the purity of DNA (normal value, ≈1.8). Lower 260:280 ratio may indicate the presence of protein, phenol, and other contaminants (absorbance at 280 nm). A secondary measurement of nucleic acid purity is the ratio of sample absorbance at 260 nm and 230 nm (normal range, 1.8–2.2). A lower 260:230 ratio may indicate the presence of co‐purified contaminants, such as salts (absorbance at 230 nm).

### 2.6 DNA quantification on agarose gel

The quality of the DNA samples was checked in 0.6% agarose gel electrophoresis and the amount of DNA in each sample was quantified using MassRuler High Range Forward and Reverse DNA Ladder (Fermentas Life Sciences, Waltham, MA) as markers. Referring to nanodrop quantification results, we took an amount of solution corresponding to 30 ng of DNA. If necessary, we eluted DNA in distilled water to obtain a final concentration of 30 ng/μl. The DNA samples (1 μl) were loaded with bromophenol blue loading dye (6×) in agarose gel. The gel is prepared by dissolving an amount equal to the 0.6% of agarose powder in TAE (tris‐acetate‐EDTA) buffer (1×) with the addition of ethidium bromide (EtBr) that intercalates DNA and makes it visible under UV light. A DNA marker with fragments of known length and concentration is run through the gel at the same time as the samples. We used MassRuler High Range Forward and Reverse DNA Ladder (Fermentas Life Sciences) as markers. Electrophoresis is carried out at 100 mV.

### 2.7 Polymerase chain reaction

The primer pair was designed according to standard criteria (Caracausi, Piovesan, Vitale, & Pelleri, 2017; Sharrocks, 1994).

**Figure 1:**
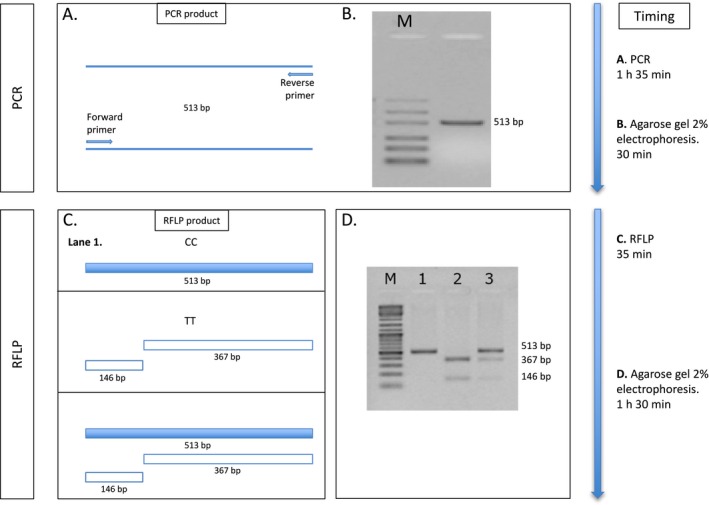
Sequence of polymerase chain reaction (PCR) (a, b) and restriction fragment length polymorphism (RFLP) (c, d) reactions for *MTHFR* C677T polymorphism analysis with a timeline indicating the time each step will take. (b) The marker used was MassRuler Low Range Reverse DNA Ladder (Fermentas Life Sciences, Waltham, MA, USA); (c) CC: wild‐type homozygote; TT: homozygote for the polymorphism; CT: heterozygote for the polymorphism. (d) The marker used was GeneRuler DNA Ladder Mix (Thermo Fisher Scientific, Waltham, MA, USA). Gel images show inverted colors

Each 50 µl PCR reaction contained 2 U of Platinum Taq DNA Polymerase (Invitrogen; Thermo Fisher Scientific), 5 µl of PCR buffer (10×), 50 mM of MgCl_2_ (final 1.5 mM), 10 mM dNTPs mix (final 0.2 mM), 0.2 µM of each primer, and 150 ng of genomic DNA template. The mixture was denatured at 94°C for 2 min and the PCR was performed for 35 cycles in a thermocycler (GenePro TC‐E‐48D, Bioer Technology, Hangzhou, China) under the following conditions: denaturation at 94°C for 30 s, annealing at 61°C for 30 s, and extension at 72°C for 30 s. The final extension cycle of 72°C was for 7 min (Figure 1a,b).

PCR products (3 µl) were loaded in 2% agarose gel with addition of EtBr to confirm the correct amplicon size (513 bp). Electrophoresis was carried out at 100 mV.

Quantification of the PCR‐products with a concentration marker is essential for the following steps and is achieved by matching the intensity of sample bands to marker bands, in this case we used MassRuler Low Range Reverse DNA Ladder (Thermo Scientific; Thermo Fisher Scientific) (Figure 1b).

### 2.8 Restriction fragment length polymorphism

For RFLP reaction, we used Anza^TM^ 71 *Hin*f I (Thermo Scientific; Thermo Fisher Scientific). 20 U (1 µl) of this enzyme is able to digest 0.2‐1 µg of PCR product in 15 min. Each 20 µl mixture reaction was prepared with 20 U (1 µl) of *Hin*f I enzyme, 100 ng of PCR product, and Red Buffer (10×). The reaction was allowed to incubate at 37°C for 15 min in a thermocycler then the enzyme was inactivated by heating at 80°C for 20 min in accordance with the manufacturer's instructions. When *MTHFR* C677T polymorphism is present, the substitution of a C with a T creates a restriction site for *Hin*f I and the DNA‐fragment of 513 bp, previously amplified in PCR, is cut in two pieces, one of 146 bp and the other of 367 bp (Figure 1c,d).

The RFLP products (15 µl) were loaded in 2% agarose gel. GeneRuler DNA Ladder Mix was used as marker. Post‐staining with EtBr was performed after electrophoresis (100 mV) for a clear identification of different genotypes (Figure 1d).

## 3 RESULTS

### 3.1 Bibliographic search

Systematic search in PubMed retrieved 4,913 PubMed records that were examined for description and suitability of PCR primers useful to characterize *MTHFR* C677T polymorphism (Table S1), and each of the 108 distinct primer pairs identified was evaluated by the same criteria used to compare our strategy to the one of Frosst et al. (1995) (Table 1 and Table S2).

### 3.2 *MTHFR* genomic sequence analysis

There are two isoforms of the *MTHFR* transcript: NM_001330358.1 and NM_005957.4. We used GeneBase 1.1 (Piovesan et al., 2016) to summarize their different features (Table S3) and to extract the sequences of the fourth and the fifth introns of the second isoform (NM_005957.4). The C677T polymorphism maps on the exon 5 of the gene, common to the two isoforms. The two isoforms are different for the coordinates of the first exon.

### 3.3 Primer design

Using Amplify software (Engels, 1993), we selected the forward primer annealing to the bases chr21:11,796,469‐11,796,449 (Human assembly GRCh38/hg38) of the gene and the reverse primer annealing to the bases chr21:11,795,978‐11,795,957 (Human assembly GRCh38/hg38). In order to follow the standard criteria for optimal primer pair design (Caracausi et al., 2017), both primers have a content of GC between 40% and 60%, respectively: the forward has 57% of GC content, the reverse has 50%; they have the same melting temperature, Tm = 66°C; their length is between 18 and 22 nucleotides. Finally, primer‐BLAST software analysis did not find nonspecific results (Table 1).

## 4 RFLP analysis

**Figure 2:**
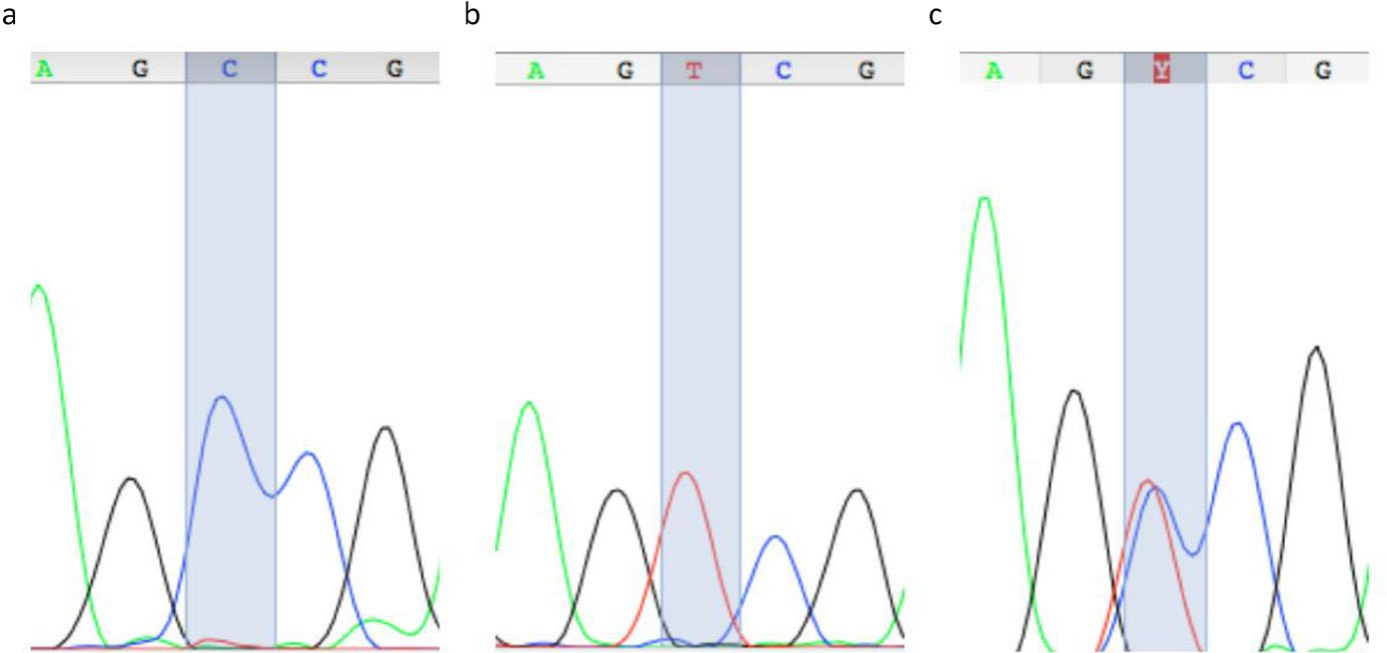
Electropherogram obtained by Sanger sequencing of RFLP control samples. (a) Wild‐type homozygote (CC); (b) C677T homozygote (TT); (c) heterozygote (CT). Blue peaks: cytosine (C); red peaks: thymine (T); grey peaks: guanine (G); green peaks: adenine (A)

We designed an amplicon of 513 bp of length that does not have restriction sites for *Hin*f I in the wild‐type isoform of the *MTHFR* gene. After the enzymatic restriction of the PCR product, the amplicon from DNA samples with C677T polymorphism is cut in two pieces, one of 146 bp and the other of 367 bp (Figure 1c and 1d). We have evaluated the robustness of our method by sequencing nine PCR products among different RFLP reactions. The electropherogram confirmed, for each sample, the PCR‐RFLP genotype (Figure 2). Automated sequencing was performed with Applied Biosystems ABI 3730 DNA.

## 5 DISCUSSION

Many kinds of approaches were used to study the *MTHFR* C677T polymorphism (Table S1), but the systematic bibliographic search performed in the present work has showed that RFLP method is still widely used throughout the world. Indeed, 2,257 out of 4,913 articles published from 1995 to 2018 used PCR‐RFLP method (45.9%) to study the MTHFR mutation and 79 articles were published in the last 2‐year period (2017–2018). We have also found that the second most used technique was Real‐time PCR described in 588 articles, 188 of them described TaqMan SNP genotyping assay sequencing. These methods and sequencing, aside from being more expensive, require test‐specific technical expertise. Furthermore, quantitative PCR (qPCR) could give false‐negative results due to mutations in the probe primer regions (Arinsburg, Shaz, Westhoff, & Cushing, 2012); genome‐wide sequencing methods might result in missing data because of inconsistency in the number of reads per sample library, the number of sites/targets per individual or the number of reads per site/target (Jiang et al., 2016). Consequently PCR‐RFLP method remains the most used technique to characterize point mutations in genomic sequences as it is relatively inexpensive and technically simple. An important step to perform a good polymorphism analysis is to design a highly specific primer pair studying the gene sequence (GeneBase 1.1 software, (Piovesan et al., 2016)), because there could be different isoforms (NM_) of the gene with a different number and sequence of exons. Gene Base 1.1 software is a full parser of the National Center for Biotechnology Information (NCBI) Gene database which generates a fully structured local database with an intuitive user‐friendly graphic interface for personal computers. It provides exon and intron sequences of each isoform, usable to design primer pairs suitable for the current polymorphism analysis; the primer pairs then need to be further analyzed:

- using reliable software (Amplify, (Engels, 1993) to check the quality of primers with regard to potential secondary structure formation (primer dimers), the GC content and the melting temperature;
- using publicly available online tools to check for biological specificity (Primer‐Blast, https://www.ncbi.nlm.nih.gov/tools/primer-blast/);
- using reliable software to check the quality of the desired amplicon, in particular the presence or absence of the restriction sites (Enzyme X 3.1).

We have shown that our original strategy of PCR‐RFLP, specifically designed to make the analysis optimal with respect to PCR primers and gel analysis fits the ideal criteria compared to the widely used strategy by Frosst et al. (1995) as well as any other PCR‐RFLP strategies proposed for the genotyping of *MTHFR* C677T polymorphism to date (Table S2).

A critical step in this protocol was to avoid a partial restriction enzyme digestion. To optimize this reaction, it was necessary to use the proper units of enzyme per μg of DNA and test reaction time.

To make sure the restriction reactions are successful, we suggest to add three controls to every digestion: one homozygous wild‐type (CC), one homozygous for the polymorphism (TT), and one heterozygous for the polymorphism (CT). The genotype of controls must be previously confirmed by Sanger sequencing.

A limit of this technique is that RNA could not be used for genotyping because both primers are designed on introns. However, it would seem to be more correct to identify the polymorphisms directly on DNA sequences because an error could occur during the transcription, for example, a mutation on the promoter in one allele could abolish RNA expression from that allele.

## CONFLICT OF INTEREST

The authors declare that they have no conflict of interest.
